# Evaluation of Molecular Point-of-Care Testing for Respiratory Pathogens in Children With Respiratory Infections: A Retrospective Case-Control Study

**DOI:** 10.3389/fcimb.2021.778808

**Published:** 2021-11-19

**Authors:** Nan Shen, Yuanjie Zhou, Yajuan Zhou, Lijuan Luo, Wenjuan Chen, Jing Wang, Ruike Zhao, Li Xie, Qing Cao, Yue Tao, Xi Mo

**Affiliations:** ^1^ Department of Infectious Diseases, Shanghai Children’s Medical Center, School of Medicine, Shanghai Jiao Tong University, Shanghai, China; ^2^ The Laboratory of Pediatric Infectious Diseases, Pediatric Translational Medicine Institute, Shanghai Children's Medical Center, School of Medicine, Shanghai Jiao Tong University, Shanghai, China; ^3^ Clinical Research Institute, School of Medicine, Shanghai Jiao Tong University, Shanghai, China

**Keywords:** pediatric respiratory infection, molecular point-of-care testing, FilmArray Respiratory Panel, antimicrobial stewardship, health economics

## Abstract

**Objectives:**

Overuse of antibiotics and antibiotic resistance are global healthcare problems. In pediatric patients with respiratory infections, viral and bacterial etiologies are challenging to distinguish, leading to irrational antibiotic use. Rapid and accurate molecular diagnostic testing methods for respiratory pathogens has been shown to facilitate effective clinical decision-making and guide antibiotic stewardship interventions in the developed regions, but its impacts on pediatric patient care in the developing countries remain unclear.

**Methods:**

In this single-center, retrospective case-control study, we compared demographics, clinical characteristics, especially microbiological findings, and antibiotic usage between pediatric patients with respiratory infection receiving FilmArray Respiratory Panel (FilmArray RP) testing and a matched routine testing control group. Our primary outcome was the duration of intravenous antibiotics treatment (DOT) during hospitalization.

**Results:**

Each group consisted of 346 children with a respiratory infection. In the FilmArray RP testing group, the DOT was shorter than that in the routine testing group (6.41 ± 3.67 days versus 7.23 ± 4.27 days; p = 0.006). More patients in the FilmArray RP testing group de-escalated antibiotic treatments within 72 hours of hospitalization (7.80%, 27/346 versus 2.60%, 9/346; p = 0.002). By contrast, fewer patients in the FilmArray RP testing group had escalated antibiotic treatments between 72 hours and seven days (7.80% versus 14.16%; p = 0.007). The cost of hospitalization was significantly lower in the FilmArray RP testing group ($ 1413.51 ± 1438.01 versus $ 1759.37 ± 1929.22; p = 0.008). Notably, the subgroup analyses revealed that the FilmArray RP test could shorten the DOT, improve early de-escalation of intravenous antibiotics within 72 hours of hospitalization, decline the escalation of intravenous antibiotics between 72 hours and seven days, and reduce the cost of hospitalization for both patient populations with or without underlying diseases.

**Conclusions:**

Molecular point-of-care testing for respiratory pathogens could help to reduce intravenous antibiotic use and health care costs of pediatric patients with respiratory infections in developing countries.

## Introduction

Respiratory infections are the leading cause of mortality in children under five years of age and represent a major burden to pediatric health in developing countries, such as China and India (Williams et al., 2002). The most common causative pathogens of severe respiratory infections are *Streptococcus pneumoniae* and *Haemophilus influenzae* or viruses such as respiratory syncytial virus (RSV) and influenza viruses in children (Bosch et al., 2013; Jain et al., 2015). However, the symptoms and signs of bacterial and viral infections are difficult to distinguish, making clinical recognition and diagnosis challenging, thus leading to excessive antibiotic administration and, consequently, increased risk of antimicrobial resistance. Children are at particular risk of this problem, because this cohort of the population has higher rates of antibiotic prescription (Hicks et al., 2015). For example, antibiotics were prescribed at about 70 million pediatric ambulatory care visits annually in the U.S., representing one-quarter of all medications dispensed to children (Hersh et al., 2011; Yonts et al., 2018). Chinese children similarly face the problem of non-rational over-prescription of antibiotics. One prospective study revealed that antibiotics are commonly overused or misused in cases of respiratory and viral infectious diseases among Chinese pediatric patients. For instance, third-generation cephalosporins were found to be the most commonly prescribed antibiotics for most respiratory tract conditions (Wang et al., 2020). Children with co-morbidities are seemed to be more susceptible to this problem of irrational use of antibiotics. The absence of early, rapid, and accurate laboratory testing for infectious pathogens is the major reason for overuse or misuse of antibiotics. The development of accurate and cost-effective rapid molecular diagnostic tests for respiratory infectious pathogens can therefore help to improve antimicrobial stewardship (AMS) (Pandolfo et al., 2021).

Nucleic acid amplification-based methods are widely used in the clinic due to their high sensitivity and specificity in the detection and identification of infectious respiratory pathogens. However, the deployment of regular PCR methods is limited by the high cost of instruments, time-consuming operations, and high technical training requirements (Zhang et al., 2011; Courtney et al., 2021). To address some of these issues, the FilmArray Respiratory Panel (FilmArray RP, BioFire Diagnostics, Utah, USA) was developed as a molecular point-of-care-testing (POCT) platform that can detect 20 viral and atypical respiratory pathogens within 1.5 hours (Poritz et al., 2011; Chan et al., 2018), and was wildly adopted in clinical management worldwide, especially in the developed regions.

No molecular multiplex POCT for respiratory pathogens including FilmArray RP has been approved for clinical use in China right now. In our previous study, we used FilmArray RP testing to describe the age groups and seasonal distributions of different pathogens in pediatric respiratory infectious patients in China (Li et al., 2018). A recently published parallel randomized controlled study showed that the use of the FilmArray RP was significantly correlated with a reduction in intravenous antibiotic use among adult patients with lower respiratory tract infection (Shengchen et al., 2019). But studies investigating the impact of FilmArray RP testing in clinical decision-making and the rational use of antibiotics in Chinese pediatric patients are still lacking. Therefore, to further investigate the impact of FilmArray RP testing on the duration and de-escalation of intravenous antibiotics in children with respiratory infections, we performed a retrospective case-control study using a historical control group of patients during the same period with no implementation of the FilmArray RP testing.

## Methods

### Study Design and Participants

This study is a single-center, retrospective case-control study conducted at the Shanghai Children’s Medical Center affiliated to Shanghai Jiao Tong University School of Medicine, Shanghai, China. The duration of therapy (DOT) and de-escalation of intravenous antibiotics were compared between patients receiving FilmArray RP v1.7 testing (12/2016-11/2017) and a matched control group (12/2015-11/2017).

We identified all patients below 18 years with a suspected respiratory infection presenting with or without fever and at least one of the following symptoms of respiratory tract illness: cough, nasal obstruction, tachypnoea, nasal flaring, or hypoxia. Patients were only included if their complete electronic medical records were available, including treatment regimens and laboratory test results.

Exclusion criteria were as follows: (1) neonates within the first 28 days after birth, (2) hospital-acquired pneumonia (Modi and Kovacs, 2020); (3) combined with other organ infection such as endocarditis, central nervous system disease, and urinary tract infection; (4) patients with hematological cancer or solid tumor receiving chemotherapy or chemoradiotherapy; (5) patients with any other conditions including major surgical procedures and trauma.

In the case group, nasopharyngeal swab (NPS) or sputum specimens obtained from patients were tested using the FilmArray RP platform, which can detect 20 pathogens, including ADV, influenza A viruses H1, 2009H1, H3 (FluA-H1, FluA-2009H1, FluA-H3) and FluB, parainfluenza virus types 1 to 4 (Para 1–4), coronaviruses 229E, HKU1, OC43, and NL63 (Cov-HKU1, NL63, 229E, OC43), human metapneumovirus (hMPV), RSV, human rhinovirus/enterovirus (Rhino/Entero), *Chlamydia pneumonia*, *Mycoplasma pneumonia*, and *Bordetella pertussis*. Sputum specimens of the control group were tested using PCR and serology methods to identify viruses and atypical bacterial pathogens.

The study was approved by the Institutional Review Board and the Ethics Committee of Shanghai Children’s Medical Center (SCMCIRB-K2017044), and written informed consent was obtained from each patient and/or their parents.

### Clinical Data Collection

The highest body temperature and lowest oxygen saturation levels were recorded from 48 hours before admission to the time of admission. These measures were performed either in the outpatient/emergency department or at home. All the other biomarker measurements were performed for all patients upon admission, including CRP, blood routine test, liver and kidney function, *etc*. No difference in the timing from admission to specimen collection or first antibiotic administration between the two groups. Electronic medical records were reviewed to obtain demographic and clinical data, including age, sex, weight, co-morbidities (including congenital heart diseases, hematological malignancies, liver diseases, genetic disorders, and organ malformations), laboratory results, antimicrobial regimens, duration of antibiotic medication, and length of hospitalization. All microbiological findings were also collected. The costs of patients’ antibiotics and hospital stays were obtained from their medical records and the hospital administration. Prophylactic antibiotics were excluded from this study.

### Outcomes

The primary outcome was the DOT during hospitalization, defined as the number of days when at least one intravenous antibiotic was administered.

The secondary outcome measurements included antiviral usage, the proportion of patients with antibiotics de-escalation within the first 72 hours, the proportion of patients with antibiotics escalation between 72 hours and seven days; the cost of intravenous antibiotics; and the cost of hospitalization. Escalation and de-escalation of antibiotics were defined as previously described (Shengchen et al., 2019). Briefly, the reduction of antibiotics, a change from intravenous antibiotics to oral antibiotics, or a change from broad-spectrum antibiotics to narrower-spectrum antibiotics (**Table S1**) were all considered de-escalations. Conversely, an increase in the types of antibiotics or a change from narrow-spectrum to broader-spectrum antibiotics were considered as an escalation. The cost of hospitalization data included four metrics: medical care (bed-day cost and doctor visit), laboratory testing (blood, pathology, and radiation tests), drugs, and other (*e.g.*, medical materials) costs. All outcomes were recorded until discharge from the hospital.

### Statistical Analysis

We used propensity score matching to compare a wide range of baseline characteristics in order to isolate any potential relationship between the outcomes and different pathogen-detection methods. In this study, we modeled the probability of testing using logistic regression and used the estimated probability as a propensity score. In addition, we included relevant baseline variables that might have affected the choice of testing methods. The following baseline characteristics were used to generate propensity scores: age, sex, body temperature, C-reactive protein (CRP), oxygen saturation, admitted to PICU, and co-morbidities. Variables were selected on the basis of clinical experience and reviews of literature. In the propensity score matching process, we adopted “greedy nearest-neighbor” matching methods without replacement. We used a 1:1 matching ratio within a caliper width of 0.05 of the standard deviation of the logit of the propensity score. The balance between groups for each covariate was evaluated by a standardized difference of less than 0.1, which was considered acceptable (Haukoos and Lewis, 2015).

Descriptive statistics are presented as frequencies with percentages, or means and standard deviations, as appropriate. Comparison between groups used either student’s *t*-test or Kruskal-Wallis rank-sum test for continuous variables, and chi-squared tests or Fisher’s exact tests for categorical variables. A 2-sided *p* < 0.05 was considered statistically significant. All statistical analyses were performed with R version 4.0.3 (http://www.R-project.org).

## Results

### Patient Population

**Figure 1:**
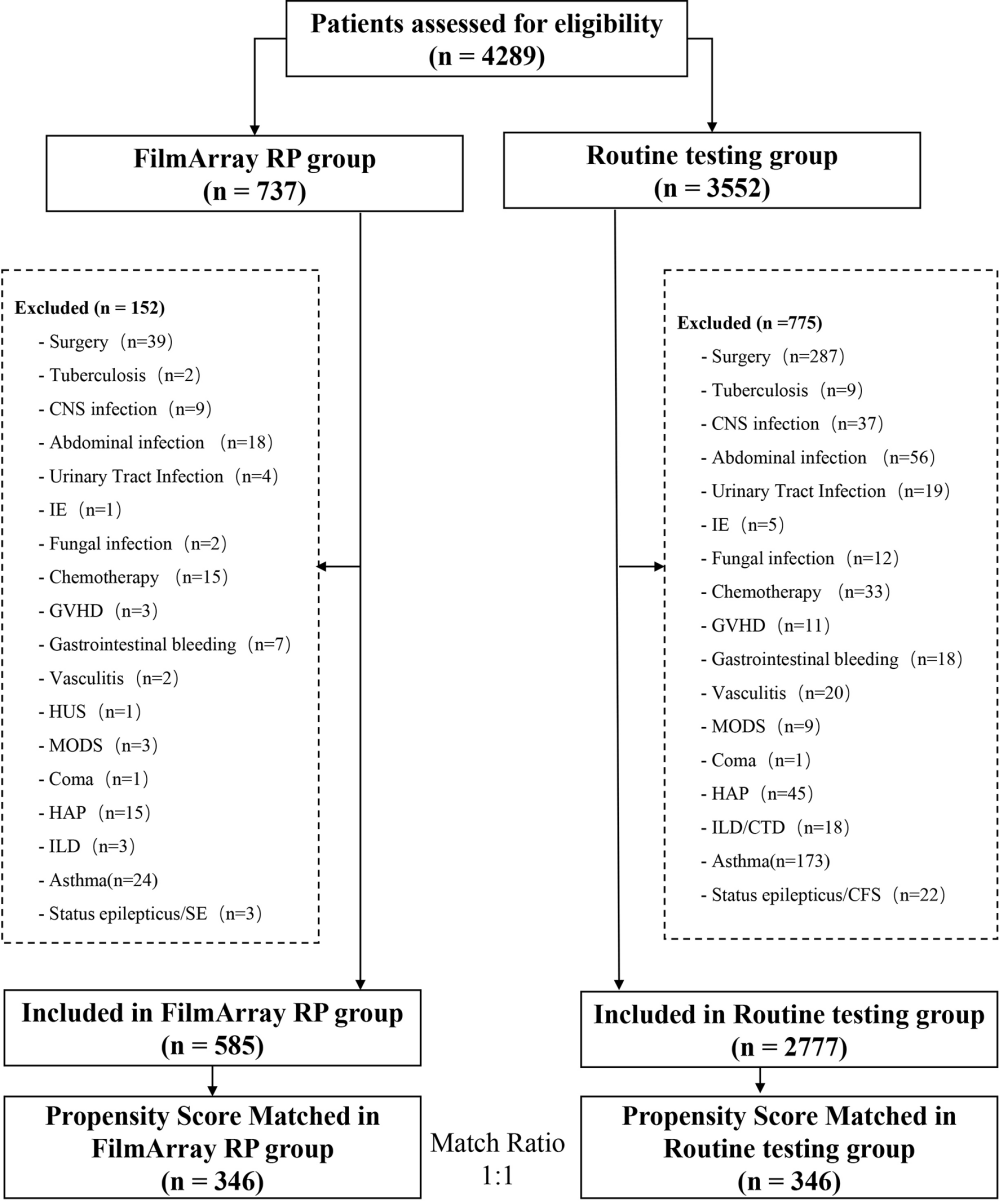
Study design and flow chart.

A total of 346 cases, matched to 346 controls, were included in this retrospective analysis based on the above inclusion and exclusion criteria (**Figure 1**). The clinical characteristics of these respiratory infection patients are shown in **Table 1**. The two groups were comparable in age, sex, symptoms on entry, admitted to PICU, and co-morbidities.

**Table 1:** Demographic and clinical characteristics of patients with respiratory infection.

Characteristic	Before Propensity Score Matching			After Propensity Score Matching		
	FilmArray RP testing group (n = 585)	Routine testing group (n = 2777)	*p*-value	FilmArray RP testing group (n = 346)	Routine testing group (n = 346)	*p*-value
Age, years	2.63 ± 2.95	2.53 ± 3.05	0.472	2.70 ± 2.89	2.48 ± 2.59	0.303
Male (%)	325 (55.56)	1590 (57.26)	0.450	183 (52.89)	191 (55.20)	0.542
**Observation**						
Weight (Kg)	13.09 ± 9.50	12.58 ± 9.95	0.259	13.11 ± 8.24	12.47 ± 7.50	0.288
Temperature (°C)	38.58 ± 1.33	38.32 ± 1.34	< 0.001	38.54 ± 1.30	38.49 ± 1.35	0.590
Respiratory frequency (breaths/min)	32.31 ± 10.00	33.54 ± 9.91	< 0.001	31.12 ± 8.95	32.22 ± 8.15	0.093
**Symptoms at entry, n (%)**						
Any respiratory symptoms						
Cough	550 (94.0)	2576 (92.8)	0.280	332 (95.95)	339 (97.98)	0.121
Dyspnea	99 (16.9)	514 (18.5)	0.361	39 (11.27)	42 (12.14)	0.723
Wheezing	200 (34.2)	992 (35.8)	0.466	118 (34.10)	139 (40.17)	0.098
Catarrhal symptoms	95 (16.2)	256 (9.2)	< 0.001	66 (19.08)	50 (14.45)	0.103
Other possible infection symptoms						
Fever (> 37.3°C)	407 (69.6)	1822 (65.6)	0.065	240 (69.36)	240 (69.36)	1.000
Diarrhea	39 (6.7)	189 (6.8)	0.896	23 (6.65)	35 (10.12)	0.100
Convulsions	7 (1.2)	63 (2.3)	0.099	3 (0.87)	5 (1.45)	0.477
**Co-morbidity (%)**			< 0.001			0.716
Without co-morbidity	462 (78.97)	1840 (66.26)		308 (89.02)	302 (87.28)	
With co-morbidity						
Congenital heart diseases	80 (13.68)	486 (17.50)		28 (8.09)	31 (8.96)	
Hematological malignancies	4 (0.68)	77 (2.77)		1 (0.29)	1 (0.29)	
Liver diseases	10 (1.71)	66 (2.38)		3 (0.87)	6 (1.73)	
Genetic disorders	6 (1.03)	41 (1.48)		5 (1.45)	3 (0.87)	
Organ malformations	5 (0.85)	88 (3.17)		1 (0.29)	1 (0.29)	
Other disease	18 (3.07)	179 (6.44)		0 (0)	2 (0.58)	
**Laboratory test**						
Procalcitonin (ng/mL)			0.197			0.933
< 0.25	399 (73.08)	2101 (75.66)		259/334 (77.54)	275/346 (79.48)	
0.25-0.49	65 (11.90)	251 (9.04)		34/334 (10.18)	33/346 (9.54)	
0.5-1.9	57 (10.44)	282 (10.15)		31/334 (9.28)	28/346 (8.09)	
≥ 2.0	25 (4.58)	143 (5.15)		10/334 (2.99)	10/346 (2.89)	
C-reactive protein (mg/L)	13.63 ± 22.59	13.31 ± 29.06	0.803	8.96 ± 10.95	8.16 ± 13.44	0.394
White blood cell count (× 10^9^/L)	10.71 ± 7.94	10.73 ± 15.14	0.980	10.07 ± 5.89	9.75 ± 4.77	0.425
Lymphocyte count-to neutrophil ratio	1.95 ± 2.73	1.87 ± 3.22	0.602	1.69 ± 2.12	1.90 ± 1.91	0.173
Haemoglobin (g/L)	118.52 ± 15.00	114.75 ± 17.56	< 0.001	118.65 ± 14.11	116.87 ± 14.26	0.100
Platelet count (× 10^9^/L)	301.21 ± 134.32	361.45 ± 158.87	< 0.001	296.42 ± 129.11	375.25 ± 149.78	< 0.001
Erythrocyte sedimentation rate (mm/H)	24.29 ± 23.05	23.75 ± 22.89	0.622	22.42 ± 21.75	23.88 ± 21.76	0.450
Albumin (g/L)	39.28 ± 4.43	37.94 ± 5.53	< 0.001	39.63 ± 4.11	38.93 ± 4.93	0.046
Alanine aminotransferase (U/L)	37.52 ± 52.32	40.28 ± 93.61	0.500	35.56 ± 42.23	32.59 ± 47.14	0.391
Creatinine (μmol/L)	3.48 ± 5.17	25.48 ± 12.20	< 0.001	3.22 ± 3.71	23.47 ± 10.12	< 0.001
Blood urea nitrogen (mmol/L)	26.69 ± 7.70	3.83 ± 2.80	< 0.001	27.05 ± 7.50	17.80 ± 13.29	< 0.001

All patients in the FilmArray RP testing group were tested using FilmArray RP, while patients in the routine testing group were tested using PCR and serological methods to identify viruses and atypical bacterial pathogens. Details of the frequency and types of pathogens detected in the two groups are presented in **Table 2**. At least one respiratory pathogens of interest were detected in 388 patients, including 279 (80.64%) in the FilmArray RP testing group and 109 (31.50%) in the routine testing group. Moreover, much more mixed infections, in which two or more pathogens were detected in the FilmArray RP testing group, occurred in 73 (26.16%) of the 346 patients. In contrast, multiple pathogens were detected in only 11 (3.18%) out of all patients in the routine testing group. Therefore, FilmArray RP testing not only provides detection for more pathogens (20 versus 7), but also had higher sensitivity than that of conventional methods, except for *Mycoplasma pneumoniae* which was detected by both PCR and serological methods in routine clinic testing.

**Table 2:** Infectious pathogens detected in patients.

Detected pathogens	FilmArray RP testing group (n = 346)	Routine testing group (n = 346)	*p*-value
**Types of pathogens detected**			
Influenza A virus (%)	14 (4.05)	2 (1.20)	0.084
Influenza B virus (%)	24 (6.94)	1 (0.60)	0.002
Respiratory syncytial virus (%)	61 (17.63)	14 (9.33)	0.018
Adenovirus (%)	38 (10.98)	2 (1.20)	< 0.001
Parainfluenza Virus type1-4 (%)	24 (6.94)	6 (4.08)	< 0.001
Human Rhinovirus/Enterovirus (%)	88 (25.43)	37 (15.55)	0.004
Human Metapneumovirus (%)	19 (5.49)	N/A*	–
Coronavirus HKU1, NL63, 229E, OC43 (%)	16 (4.62)	N/A*	–
Bordetella pertussis	29 (8.38)	N/A*	–
Mycoplasma pneumoniae	50 (14.45)	61 (18.15)	0.190
**Number of pathogen detected**			< 0.001
No pathogen detected (%)	67 (19.36)	237 (68.50)	
Single pathogen detected (%)	206 (59.54)	98 (28.32)	
Multiple pathogens detected (%)	73 (21.10)	11 (3.18)	

*N/A means the pathogens were not routinely detected in clinic.

### Outcomes

**Figure 2:**
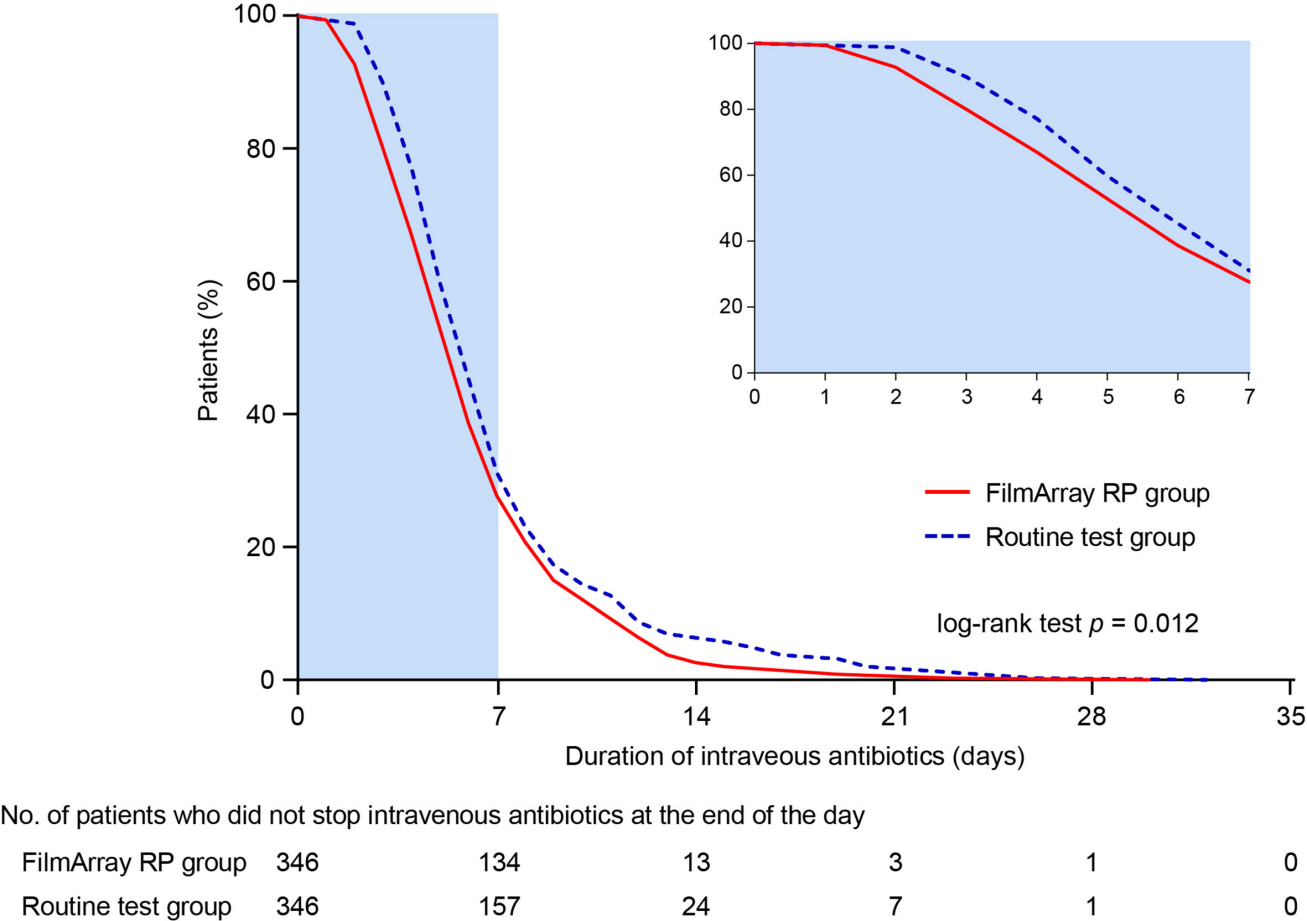
Time to withdrawal of intravenous antibiotics (case-to-treat analysis).

The mean DOT in the FilmArray RP testing group was significantly shorter than that in the routine testing group (6.41 ± 3.67 days versus 7.23 ± 4.27 days; difference -0.83 days, 95% CI -1.42 to -0.23 days; *p* = 0.006) (**Table 3**). In addition, intravenous antibiotic treatments were halted earlier in the FilmArray RP testing group compared to the routine testing group (*p* = 0.012; log-rank test) (**Figure 2**). More patients in the FilmArray RP testing group de-escalated antibiotic treatments within 72 hours than in the routine testing group (7.80%, 27/346 versus 2.60%, 9/346; *p* = 0.002). By contrast, fewer patients in the FilmArray RP testing group had an escalation of their antibiotic regimen between 72 hours and seven days (7.80% 9/346 versus 14.16%, 49/346; *p* = 0.007) than in the routine testing group. Notably, antivirals were used more often in the FilmArray RP testing group (34.10%, 118/346 versus 10.12%, 35/346; *p* < 0.001), especially for oseltamivir (8.96%, 31/346 versus 1.73%, 6/346; *p* < 0.001), indicating a more rational prescription of antimicrobial drugs.

**Table 3:** Primary and secondary outcomes among total matched population.

Clinical Outcomes	FilmArray RP testing group (n = 346)	Routine testing group (n = 346)	Differences (95% CI)	*p*-value
**Primary outcome**				
Duration of intravenous antibiotics (days)	6.41 ± 3.67	7.23 ± 4.27	-0.83 (-1.42, -0.23)	0.006
**Secondary outcome**				
De-escalation within the first 72 h (%)	27 (7.80)	9 (2.60)	–	0.002
Escalation between 72 h and 7 days (%)	27 (7.80)	49 (14.16)	–	0.007
Cost of intravenous antibiotics ($)*	109.84 ± 177.12	144.39 ± 310.31	-34.54 (-72.26, 3.17)	0.073
Cost of hospitalization ($)*	1413.51 ± 1438.01	1759.37 ± 1929.22	-345.86 (-599.84, 91.88)	0.008

*Exchange rate: US dollar: Chinese yuan = 1: 6.5.

The mean cost of intravenous antibiotics was less in the FilmArray RP testing group than in the routine testing group ($ 109.84 ± 177.12 versus $ 144.39 ± 310.31; difference -34.54, 95% CI -72.26 to 3.17; *p* = 0.073), despite statistical significance was not reached. Additionally, the mean cost of hospitalization was significantly lower in the FilmArray RP testing group ($ 1413.51 ± 1438.01 versus $ 1759.37 ± 1929.22; difference -345.86, 95% CI -599.84 to -91.88; *p* = 0.008). Taken together, these results indicated that the FilmArray RP testing was associated with shorter intravenous antibiotic treatment periods, more frequent antibiotics de-escalation in the first 72 hours, less frequent escalation between 72 hours and 7 days, and lower overall hospital costs compared to patients receiving routine tests.

### Subgroup Analysis of Clinical Outcomes by Co-Morbidity Status

Subgroup analyses were conducted according to whether the patients had underlying disease. A total of 308 cases matched to 302 controls, without underlying diseases, were included in the no co-morbidity subgroup. Details of the frequency and types of pathogens detected in the subgroup analyses are presented in **Table S2**. Similar to the total population analyzed above, the mean DOT of patients who received the FilmArray RP test was significantly shorter than those who received routine tests (6.01 ± 3.04 days versus 6.73 ± 3.59 days; difference -0.72 days, 95% CI -1.25 to -0.19 days; *p* = 0.008). In addition, more patients in the FilmArray RP testing group de-escalated antibiotic treatments within 72 hours (7.79%, 24/308 versus 2.98%, 9/302; p = 0.009), and fewer patients in the FilmArray RP testing group escalated antibiotic treatments between 72 hours and seven days than in the routine testing group (7.79%, 24/308 versus 13.58%, 41/302; *p* = 0.021). The mean cost of intravenous antibiotics was comparable between the patients in the two groups, but the mean cost of hospitalization in the FilmArray RP testing group was significantly lower than in the routine testing group ($ 1253.20 ± 754.50 versus $ 1505.47 ± 1078.05; difference -1639.73, 95% CI -2599.95 to -679.52; *p* < 0.001) (**Table 4**).

**Table 4:** Subgroup analysis of clinical outcomes under different co-morbidity status at baseline.

Clinical Outcomes	FilmArray RP testing group	Routine testing group	Differences (95% CI)	*p*-value
**No co-morbidity group**				
Number of patients	308	302	–	–
Duration of intravenous antibiotics (days)	6.01 ± 3.04	6.73 ± 3.59	-0.72 (-1.25, -0.19)	0.008
De-escalation within the first 72 h (%)	24 (7.79)	9 (2.98)	–	0.009
Escalation between 72 h and 7 days (%)	24 (7.79)	41 (13.58)	–	0.021
Cost of intravenous antibiotics ($)*	96.07 ± 110.28	109.24 ± 209.01	-85.61 (-257.86, 86.65)	0.329
Cost of hospitalization ($)*	1253.20 ± 754.50	1505.47 ± 1078.05	-1639.73 (-2599.95, -679.52)	< 0.001
**Co-morbidity group**				
Number of patients	38	44	–	–
Duration of intravenous antibiotics (days)	9.61 ± 6.08	10.68 ± 6.52	-1.08 (-3.86,1.71)	0.444
De-escalation within the first 72 h (%)	3 (7.89)	0 (0.00)	–	0.058
Escalation between 72 h and 7 days (%)	3 (7.89)	8 (18.18)	–	0.234
Cost of intravenous antibiotics ($)*	221.46 ± 420.97	385.61 ± 631.32	-1033.96 (-2625.99, 492.07)	0.177
Cost of hospitalization ($)*	2712.84 ± 3551.11	3502.05 ± 4262.16	-5129.84 (-16442.97, 6183.30)	0.370

*Exchange rate: US dollar: Chinese yuan = 1: 6.5.

For patients with underlying diseases, a total of 38 cases matched to 44 controls were included in the co-morbidity status subgroup. The underlying diseases consisted of congenital heart diseases, hematological malignancies, liver diseases, genetic disorders, and organ malformations. Although none of the five outcome indicators reached statistical significance between the FilmArray RP and routine testing groups, possibly because of the small population size, the FilmArray RP testing group still showed the same trend as the whole population. The mean DOT was one day shorter in the FilmArray RP testing group, and the mean costs of intravenous antibiotics and hospitalization were also about $ 164 and $ 790 less, respectively. Notably, three patients (7.89%) in the FilmArray RP testing group de-escalated antibiotic treatments within 72 hours, compared with no patient (0.00%) in the routine testing group. Also, only three patients (7.89%) in the FilmArray RP testing group escalated antibiotic treatments between 72 hours and seven days, while eight patients (18.18%) in the routine testing group had an escalation of their antibiotic regimen (**Table 4**).

## Discussion

The irrational administration of antibiotics for respiratory infectious diseases and the resulting emergence of antibiotic resistance have been recognized as global problems and highlight the urgent need to reduce unnecessary antibiotics usage and improve AMS (Hutchings et al., 2019). One approach to resolve these problems is to increase the diagnostic accuracy of current tests for respiratory pathogens, especially in children whose causing pathogens for respiratory infections are mainly viruses (de Vries et al., 2020). In this study, we showed that molecular POCT for respiratory pathogens using the FilmArray RP platform could shorten the DOT, improve early de-escalation of intravenous antibiotics, and reduce the cost of intravenous antibiotics and hospitalization in Chinese pediatric in-patients with respiratory infections. Notably, we also found that the FilmArray RP platform would facilitate the rational use of antibiotics in pediatric patients with or without underlying diseases.

The efficacy of FilmArray RP testing has been evaluated in multiple retrospective and prospective studies in both adults and children with respiratory infections. The detection of atypical pathogens and respiratory viruses using FilmArray RP was shown to outperform currently used conventional methods (Pierce et al., 2012; Piralla et al., 2014; Huang et al., 2018). Furthermore, compared to routine tests, which usually need 1-2 days to report results, the mean turnaround time (TAT) of FilmArray RP was approximately 4 hours. The improved TAT and detection rates for respiratory viruses could potentially reduce the irrational prescription of antibiotics. A recent study compared trends in antibiotics prescription among influenza patients diagnosed using rapid molecular POCT or standard multiplex PCR (Au Yeung et al., 2021). The more rapid testing method was associated with a reduced likelihood of antibiotic commencement (51% versus 67% of patients, respectively; *p* < 0.01) and more frequent commencement of oseltamivir (69% versus 56%; *p* = 0.02), which is consistent with the results of our study. We also confirmed that positive test results for influenza or RSV could prompt earlier discontinuation of antibiotics. However, rapid diagnostic testing methods for infectious pathogens cannot completely eliminate the irrational use of antibiotics since the clinical practice of continuing antibiotic regimens until patients obtain negative bacterial results is understandable, if not intuitive, especially in patients with respiratory infections. Accumulating evidence suggests that only a combination of interventions that include continuing education programs, changes in public health policy, healthcare information system reminders, and accurate laboratory testing for infectious pathogens can promote the rational use of antibiotics (Yao et al., 2020).

In this study, we focused on the DOT as well as the costs of antibiotics and hospitalization to evaluate molecular POCT for pediatric cases of respiratory infectious disease. Nevertheless, the specific outcomes used to evaluate these tests should be defined by local need (*i.e.*, disease prevalence). For example, several studies have demonstrated the positive effects of implementing molecular POCT through antimicrobial usage indicators (duration of therapy, appropriateness of antibiotic prescription, change/cessation of antimicrobials) and clinical indicators (length of stay, mortality) (Shengchen et al., 2019; Kitano et al., 2020; Saarela et al., 2020). However, little or no data has been reported on the use of other outcomes, such as the effects of molecular POCT on multidrug-resistant bacterial infection rates/antibiotic susceptibility. Therefore, rigorous and systematic analysis is urgently needed to determine which outcomes are reliable for comparing POCT methods, especially among pediatric patients.

We also paid attention to the impact of molecular POCT on reducing the usage of antibiotics and the cost of hospitalization in the subgroup patients with co-morbidity status, such as congenital heart disease, due to relatively high antibiotic consumption in these children. In this study, we found that more patients had respiratory viruses (*i.e.*, ADV, RV, and RSV) detected by FilmArray RP, suggesting that targeting the molecular tests to patients with co-morbidities would probably be more beneficial. Furthermore, the subgroup analyses revealed that the FilmArray RP test could shorten the DOT, improve AMS, and reduce the cost of hospitalization for patients with co-morbidity status, although the differences did not reach statistical significance, which is probably due to the small sample size.

It warrants mention that the cost of FilmArray RP testing, which is always a major consideration in developing counties and regions, was not included among the total hospitalization costs in this study, since the platform was not yet commercially available in China. Our study indicated that the hospitalization costs for the FilmArray RP testing group would be lower than, or at least equal to, that of the routine testing group if the FilmArray RP test cost is less than $ 345.86. With the development of technology, it is conceivable that the FilmArray RP test or other molecular POCT can achieve at a much lower cost in the future and become a routine clinical testing method in more regions. Therefore, in addition to reducing the cost for patients, these molecular POCT might give more value to more patients with shorter antibiotic treatment periods and more effective treatments, which also profoundly affects AMS.

This study comes with several limitations. First, this was a retrospective and single-center study; prospective multicenter studies could provide stronger statistical support for our conclusions in future work. Second, the different impacts of molecular POCT on AMS at different key decision nodes (*e.g.*, the need for antibiotics initiation, on-treatment, and de-escalation/cessation of treatment) were not evaluated. Third, the study was case-control design in nature, which is usually prone to confounding bias, potentially leading to an inflated estimation of the diagnostic performance.

In conclusion, the results shown here indicate that molecular POCT for respiratory pathogens could help to reduce intravenous antibiotic use and health care costs in Chinses pediatric patients with respiratory infections. Broader application of such testing may help to improve AMS and alleviate the current growing problem of antibiotic resistance.

## Data Availability Statement

The original contributions presented in the study are included in the article/**Supplementary Material**. Further inquiries can be directed to the corresponding authors.

## Ethics Statement

The studies involving human participants were reviewed and approved by The Institutional Review Board and the Ethics Committee of Shanghai Children’s Medical Center (SCMCIRB-K2017044). Written informed consent to participate in this study was provided by the participants’ legal guardian/next of kin.

## Author Contributions

XM, YT, and QC conceived and designed this study. NS and YuZ collected the clinical data. NS, XM, YT, and QC wrote the manuscript. YaZ, LL, WC, JW, and RZ helped analyze the data. LX helped design this study and performed all the statistical analysis. All authors contributed to the article and approved the submitted version.

## Funding

This work was funded by Shanghai Key Laboratory of Clinical Molecular Diagnostics for Pediatrics (20dz2260900), Shanghai Key Laboratory of Emergency Prevention, Diagnosis and Treatment of Respiratory Infectious Diseases (20dz2261100), Scientific and Technology Commission of Shanghai Municipality (20Y11903600), Shanghai Municipal Health Commission (2019SY049), and Collaborative Innovation Center for Translational Medicine at Shanghai Jiao Tong University School of Medicine (TM201927).

## Conflict of Interest

The authors declare that the research was conducted in the absence of any commercial or financial relationships that could be construed as a potential conflict of interest.

## Publisher’s Note

All claims expressed in this article are solely those of the authors and do not necessarily represent those of their affiliated organizations, or those of the publisher, the editors and the reviewers. Any product that may be evaluated in this article, or claim that may be made by its manufacturer, is not guaranteed or endorsed by the publisher.
