# Supplementary material for: Evaluation of Molecular Point-of-Care Testing for Respiratory Pathogens in Children With Respiratory Infections: A Retrospective Case-Control Study
Source: Front Cell Infect Microbiol. 2021 Nov 19;11:778808. doi: 10.3389/fcimb.2021.778808 (PMC8640230; doi:10.3389/fcimb.2021.778808)
Supplement: Supplementary file 1 [file DataSheet_1.docx]

**Table S1. Definition of antibiotics de-escalation**

| **Antibiotics de-escalation** |  | |
| --- | --- | --- |
| Reduction of antibiotic types | A+B to A/B or to discontinuation |  |
| Intravenous antibiotics | to oral antibiotics |  |
| Broader-spectrum antibiotics to narrower spectrum antibiotics | Broader-spectrum antibiotics: Levofloxacin, Types III or IV generation cephalosporin, Carbapenem, β-lactamase/β-lactamase-inhibitors  Narrower spectrum antibiotics: Macrolide, Types I or II generation cephalosporins, Penicillins |  |

**Table S2. Subgroup analysis of infectious pathogens detected in patients**

|  | **Co-morbidity group** | | |  | **No co-morbidity group** | | |
| --- | --- | --- | --- | --- | --- | --- | --- |
| **Types of pathogens detected** | **FilmArray RP**  **testing group (n=38)** | **Routine testing group**  **(n=44)** | ***p*-value** |  | **FilmArray RP**  **testing group (n=308)** | **Routine testing group**  **(n= 302)** | ***p*-value** |
| Influenza A virus | 0.00%, 0/38 | 0.00%, 0/19 | - |  | 4.55%, 14/308 | 1.36%, 2/147 | 0.085 |
| Influenza B virus | 0.00%, 0/38 | 0.00%, 0/19 | - |  | 7.79%, 24/308 | 0.68%, 1/147 | 0.002 |
| Respiratory syncytial virus | 26.32%, 10/38 | 11.11%, 2/18 | 0.195 |  | 16.56%, 51/308 | 9.09%, 12/132 | 0.040 |
| Adenovirus | 13.16%, 5/38 | 0.00%, 0/19 | 0.098 |  | 10.71%, 33/308 | 1.36%, 2/147 | < 0.001 |
| Parainfluenza Virus type1-4 | 5.26%, 2/38 | 11.76%, 2/17 | 0.067 |  | 7.14%, 22/308 | 3.08%, 4/130 | 0.006 |
| Human Rhinovirus/Enterovirus | 26.32%, 10/38 | 8.00%, 2/25 | 0.070 |  | 25.32%, 78/230 | 16.43%, 35/213 | 0.015 |
| Human Metapneumovirus | 0.00%, 0/38 | N/A* | - |  | 6.17%, 19/308 | N/A* | - |
| Coronavirus HKU1, NL63, 229E, OC43 | 7.89%, 3/38 | N/A* | - |  | 4.22%, 13/308 | N/A* | - |
| Bordetella pertussis | 2.63%, 1/38 | N/A* | - |  | 9.09%, 28/308 | N/A* | - |
| Mycoplasma pneumoniae | 5.26%, 2/38 | 2.44%, 1/41 | 0.512 |  | 15.58%, 48/308 | 20.01%, 60/295 | < 0.001 |

*N/A means the pathogens were not routinely detected in clinic.
